# Is Human Chorionic Gonadotropin Trigger Beneficial for Natural Cycle Frozen-Thawed Embryo Transfer?

**DOI:** 10.3389/fmed.2021.691428

**Published:** 2021-10-14

**Authors:** Dan-Dan Gao, Li Li, Yi Zhang, Xiao-Xuan Wang, Jing-Yan Song, Zhen-Gao Sun

**Affiliations:** ^1^College of Traditional Chinese Medicine, Shandong University of Traditional Chinese Medicine, Jinan, China; ^2^The First Clinical College, Shandong University of Traditional Chinese Medicine, Jinan, China; ^3^Reproductive and Genetic Center of Integrated Medicine, The Affiliated Hospital of Shandong University of Traditional Chinese Medicine, Jinan, China

**Keywords:** human chorionic gonadotropin, modified natural cycle, natural cycle, propensity score matching, frozen-thawed embryo transfer (FET)

## Abstract

**Objective:** The aim of this study is to investigate, in ovulatory patients, whether there is a difference in reproductive outcomes following frozen-thawed embryo transfer (FET) in natural cycles (NC) compared to modified natural cycles (mNC).

**Methods:** This retrospective cohort study, performed at the public tertiary fertility clinic, involved all infertile patients undergoing endometrial preparation prior to FET in NC and mNC from January, 2017 to November, 2020. One thousand hundred and sixty-two patients were divided into two groups: mNC group (*n* = 248) had FET in a NC after ovulation triggering with human chorionic gonadotropin (hCG); NC group (*n* = 914) had FET in a NC after spontaneous ovulation were observed. The primary outcome was live birth rate. All pregnancy outcomes were analyzed by propensity score matching (PSM) and multivariable logistic regression analyses.

**Results:** The NC group showed a higher live birth rate [344/914 (37.6%) vs. 68/248 (27.4%), *P* = 0.003; 87/240 (36.3%) vs. 66/240 (27.5%), *P* = 0.040] than the mNC group before and after PSM analysis. Multivariable analysis also showed mNC to be associated with a decreased likelihood of live birth compared with NC [odds ratio (OR) 95% confidence interval (CI) 0.71 (0.51–0.98), *P* = 0.039].

**Conclusion:** For women with regular menstrual cycles, NC-FET may have a higher chance of live birth than that in the mNC-FET cycles. As a consequence, it's critical to avoid hCG triggering as much as possible when FETs utilize a natural cycle strategy for endometrial preparation. Nevertheless, further more well-designed randomized clinical trials are still needed to determine this finding.

## Introduction

The last three decades have seen a growing trend toward embryo vitrification technology, frozen-thawed embryo transfer (FET) has become an indispensable part of assisted reproductive technology (ART) (1, 2). The practice of FET has been bolstered by substantial advancements in vitrification technology and the reported excellent pregnancy and neonatal results (3, 4). When there are enough embryos after fresh embryo transfer cycles, when a freeze-all strategy is utilized after a gonadotrophin-releasing hormone (GnRH) agonist trigger in GnRH antagonist protocols for patients at risk of ovarian hyperstimulation syndrome (OHSS), when late-follicular progesterone rise occurs, and when there is embryo-endometrial asynchrony, FET is accomplished (5).

Endometrial preparation is a pivotal link in FET technology, of which natural cycle (NC), modified natural cycle (mNC) and hormone replacement cycle are commonly used endometrial preparation protocols before frozen-thawed embryo transfer in clinical practice. There is no agreement to date about what procedure leads to best pregnancy outcomes (6–8). For patients with normal ovulation, NC is often used to prepare the endometrium prior to FET, of which NC is closer to the state of natural conception due to the avoidance of exogenous hormones to prime the endometrium. This is in line with the requirements of embryo implantation, has the advantages of less drug use and easier to be accepted by patients. Hence, it has become a common method of endometrial preparation (9). Nevertheless, NC-FET requires close monitoring of follicular development, endometrial thickness, and hormone levels, while mNC-FET only requires regular monitoring of ultrasound and serum luteinizing hormone (LH) levels (10). Because the endogenous LH is not easy to be measured accurately and the follicle may not be ruptured, hCG is often used to simulate the endogenous LH peak to induce ovulation. Due to the fact that hCG has a lengthy half-life and a sustained luteotropic effect in the early luteal phase for up to 7 days after administration (11), luteal phase support (LPS) might not be needed in mNC (10, 12). The use of exogenous estrogen in hormone replacement cycles can achieve ideal endometrial thickness in most patients, and it is also convenient to control the timing of embryo transfer. However, long-term use of exogenous estrogen and progesterone may increase the risk of thrombosis and uterine fibroid recurrence (13). For patients with regular menstruation and normal ovulation, the natural cycle is the safest and most economical endometrial preparation program because it avoids the use of exogenous hormones. The best-individualized approach to endometrium preparation for FET is still a matter of dispute, considering the growing interest in FET and tailored approaches to reproductive medicine (14, 15). Some researchers pay attention to the effect of hCG on the pregnancy outcomes of FET in natural cycle, but there is no unified answer (16–18).

Successful embryo implantation requires a complex and efficient interaction between the embryo and the endometrium (19). Embryos and endometrium must be fully synchronized within a certain time frame, which is the notion of “implantation window period” or “endometrial receptivity” (20). However, the question about the optimal method for the preparation of endometrium remains unanswered (21). As a result, we decided to perform this retrospective cohort study to compare the pregnancy outcomes of NC and mNC patients undergoing endometrial preparation for FET.

## Materials and Methods

### Population and Study Design

In our retrospective cohort study, the study population included women undergoing IVF treatment at the Reproductive and Genetic Center of Integrated Medicine of the affiliated Hospital of Shandong University of Traditional Chinese Medicine (TCM) in the people's Republic of China from January 2017 to November 2020. All patients were followed up for at least 1 year, and the data were extracted from the electronic medical record system. The study was approved by the Reproductive Ethics Committees of the Affiliated Hospital of Shandong University of TCM (Ref no. SDTCM-E20.12.1501) and was performed in accordance with relevant guidelines and regulations. Informed consent was abandoned because of the retrospective nature of the study. Moreover, the recognition of participants by the research data was absolutely omitted.

Patients who meet the following inclusion criteria are considered qualified: 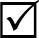 Women who have had IVF embryo freezing cycles; 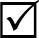 Having at least 1 day three vitrified embryos for frozen-thawed embryo transfer; 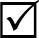 The first FET cycle; 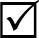 Regular menstrual cycles (25–35 days).

Patients meeting the following exclusion criteria must be disqualified: 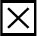 Undergoing the second FET or further frozen cycles; 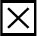 Being older than 40 years at the time of embryo vitrification; 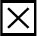 Sever endometriosis; 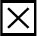 History of recurrent pregnancy loss; 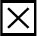 Pre-implantation genetic diagnosis/screening cycle; 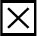 Uterine pathology; 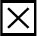 Cycles canceled owing to embryo thawing and survival failures.

### Controlled Ovarian Stimulation Protocol

Each participant had had IVF/ICSI treatment as clinically indicated. Additionally, a fixed GnRH antagonist (GnRH-ant) regimen was used with 150–250 IU/day of recombinant FSH (Cetrorelix; Merck Serono, Darmstadt, Germany) (Gonal-F, Merck-Serono, Lyon, France). Moreover, doses of gonadotropin were determined based on the characteristics of each individuals. After triggering with recombinant hCG (250 μg, Ovitrelle®, Merck), oocyte retrieval was done under transvaginal ultrasound guidance 34–36 h later, followed with standard IVF/ICSI as previously reported (22). Following oocyte retrieval, fresh embryo transfer and vitrification of surplus excellent available embryos were performed, or a freeze-all strategy was used when clinically recommended. During controlled ovarian stimulation (COS), routine monitoring is needed. Vaginal ultrasonography (to evaluate endometrial thickness and follicular growth) and blood hormone tests are routinely performed [including estradiol (E_2_), progesterone (P_4_) and LH plasma levels].

The choice of embryos for vitrification was expected to focus on the inclusion of no <6 blastomeres with ≤ 20% fragmentation. Embryos that presented a fragmentation rate between 20 and 50% were vitrified only when they had reached the 8-cell stage on Day 3. The applied vitrification and warming procedure have been described in detail previously (23).

### Endometrial Preparation Protocols and FET

The attending doctors choose the kind of endometrial preparation for each patient at their discretion. Generally, individuals who ovulated regularly were given NC or mNC cycles. Additionally, NC-FET limits the capacity to manage the workload in the ART laboratory, since the reproductive center must be prepared to conduct embryo transfer on any given day of the week. In what is known as mNC-FET, ovulation may be induced with an hCG injection to enhance, at least partly, control over FET operation planning.

No medical intervention occurs in the NC-FET group. Transvaginal ultrasonography was used to assess follicle development beginning on cycle day 8. When the diameter of the dominant follicle reached 14 mm, patients were followed daily to identify ovulation and LH surge, defined as a blood hormone level that was at least 1.8 times greater than the most recently recorded serum value and continued to increase thereafter (24). Whereas, blood tests were obtained the day following the LH surge to confirm ovulation, a reduction in serum *E*_2_ and a concurrent increase in serum *P*_4_ > 1.5 ng/ml was required. Daily transvaginal ultrasound was used to establish the day of spontaneous ovulation, and 3 days later FET was planned for cleavage-stage embryos.

On days 8–10 of the menstrual cycle, patients with mNC-FET had transvaginal ultrasonography. Follicular development was assessed using transvaginal ultrasonography and serum LH levels. When the dominant follicle attained an average diameter of > 17 mm and the serum LH concentration was 20 IU/L, highly pure urinary hCG (10,000 IU, Pregnyl®) was given to induce oocyte ovulation. Transvaginal ultrasonography was performed daily before to ovulation when serum LH levels exceeded 20 IU/L. The day of ovulation was confirmed by vaginal ultrasonography, after which FET was scheduled 3 days later for cleavage-stage embryos. after which FET was scheduled 3 days later for cleavage-stage embryos. For the purpose of synchronization between embryos and endometrium, 2 days after the hCG trigger corresponds to the day of oocyte retrieval in the fresh IVF-ET cycles (21).

FET was planned 5 days after hCG administration for cleavage stage embryos. Day 3 of the cleavage stage of vitrified embryos was warmed in the morning, the day before embryo transfer. Once warming was achieved, the viability of embryos was measured morphologically. Chinese law allows the use of a maximum of two embryos per FET. Prior to beginning the procedure, the clinician determined that one or two embryos should be transferred. In neither of the groups was additional LPS administered. A pregnancy test was performed 14 days after FET.

### Study Endpoints and Definitions

We compared the pregnancy outcomes of the mNC and NC endometrial preparation protocols. Our primary outcome measure was live birth, which we defined as the delivery of at least one infant with breathing and heartbeat, regardless of gestational age. Positive pregnancy, defined as a serum -hCG level of at least 10 mIU/mL. After 10 gestational weeks, clinical pregnancy is defined as an intrauterine gestational sac with fetal heartbeat identified through transvaginal ultrasonography. Biochemical pregnancy loss is described as undetected pregnancy losses that are recorded only *via* a positive pregnancy test (serum-hCG level 10 mIU/mL). Clinical pregnancy loss is defined as the spontaneous loss of pregnancy before the completion of 20 gestational weeks that is recognized clinically. Ectopic pregnancy is described as a pregnancy that occurs outside the uterine cavity.

### Statistical Analysis

All data are analyzed using the SPSS software in version 26.0 (SPSS Inc., Chicago, USA). Hypotheses were tested using Chi-square statistics, Mann-Whitney U-tests, or Student *t*-tests for baseline characteristics and pregnancy outcomes according to FET endometrium preparation protocols, depending on the study question, the type and distribution of data, and the sample size. A statistically significant *P* < 0.05 was taken into account. Additionally, a propensity score matching (PSM) model was developed to balance baseline attributes disparities between the two groups. PSM is a research strategy that is often used in the social sciences to eliminate confounding bias in observational studies by combining variables linearly and compressing relevant factors into a single score (25). We selected 14 covariates to estimate the propensity score by logistic regression, including female age, body mass index (BMI), infertility type (primary or secondary), infertility duration, gravidity, parity, bFSH, initial treatment (IVF or ICSI), numbers of oocytes retrieved, total number of embryos, numbers of transferred embryos, protocol in fresh cycle (fresh embryo transfer, freeze-all strategy), good-quality embryos (yes, no) as well as endometrial thickness before FET. The NC-FET group was paired 1:1 with the mNC-FET group using the nearest-neighbor random matching algorithm. In our study, we also used binary multivariate logistic regression analysis to assess the association between endometrial preparation protocols and pregnancy outcomes after adjusting for 14 confounding variables such as female age (<37, ≥37 years), infertility duration, gravidity, parity, infertility type (primary, secondary), BMI (<25, ≥25 kg/m^2^), basic FSH (<10, ≥10 UI/L), initial treatment (IVF, ICSI), number of oocytes retrieved (≤3, 4–9, 10–15, >15), total number of embryos (<3, ≥3), number of transferred embryos, protocol in fresh cycle (fresh embryo transfer, freeze-all strategy), good-quality embryos (yes, no) and endometrial thickness before FET. We calculated crude OR and adjusted OR with 95% CI.

## Results

### Demographic Patient and ART Characteristics

Table 1 shows patient profiles before and after PSM. Concisely, a total of 1,162 FET cycles (248 cycles in mNC group and 914 cycles in NC group) were screened from our database. The PSM analysis strategy resulted in 240 matched pairs in each group. As expected, no significant between-group differences were found in post-matching analysis with regard to all baseline characteristics, including female age, BMI, infertility type and duration, gravidity, parity, basal FSH, initial treatment, oocytes retrieved, total number of embryos, transferred embryos as well as endometrial thickness before FET (*P* > 0.05; see Table 1).

**Table 1:** Demographic characteristics of two groups before and after PSM.

**Variables**	**Before PSM**			**After PSM**		
	**mNC (*N* = 248)**	**NC (*N* = 914)**	***P-*value**	**mNC (*N* = 240)**	**NC (*N* = 240)**	***P*-value**
Female age (years)	33.5 ± 4.5	32.9 ± 4.7	0.064^a^	33.5 ± 4.5	33.2 ± 4.8	*0.422^a^*
Infertility duration (years)	3.2 ± 2.2	3.2 ± 2.4	0.885^a^	3.2 ± 2.1	3.2 ± 2.4	*0.067^a^*
BMI (kg/m^2^)	23.3 ± 3.6	22.8 ± 3.3	0.051^a^	23.2 ± 3.5	22.7 ± 3.3	*0.120^a^*
Gravidity (*n*)	1 (0.2)	1 (0.2)	**0.038^c^**	1 (0.2)	1 (0.2)	*0.909^c^*
Parity (*n*)	0 (0.1)	0 (0.1)	0.087^c^	0 (0.1)	0 (0.1)	*0.998^c^*
Infertility type (*n*, %)			0.057^b^			*0.778^b^*
Primary infertility	90/248 (36.3%)	393/914 (43.0%)		90/240 (37.5%)	93/240 (38.8%)	
Secondary infertility	158/248 (63.7%)	521/914 (57.0%)		150/240 (62.5%)	146/240 (61.2%)	
bFSH (UI/L)	7.8 ± 3.2	7.8 ± 2.6	0.978^a^	7.8 ± 3.2	8.0 ± 3.1	*0.466^a^*
Initial treatment (*n*, %)			0.138^b^			*0.751^b^*
IVF	185/248 (76.4%)	722/914 (79.0%)		182/240 (75.8%)	179/240 (74.6%)	
ICSI	63/248 (25.4%)	192/914 (21.0%)		58/240 (24.2%)	64/240 (25.4%)	
Oocytes retrieved (*n*)	13.7 ± 7.9	13.4 ± 7.9	0.760^a^	13.6 ± 7.8	14.2 ± 8.5	*0.390^a^*
Total number of embryos (*n*)	4.7 ± 2.8	4.7 ± 2.9	0.836^a^	4.7 ± 2.8	4.7 ± 3.1	*0.926^a^*
Protocol in fresh cycle			0.342^b^			*0.357 ^b^*
Fresh embryo transfer	32/248 (12.9%)	140/914 (15.3%)		37/240 (15.4%)	302/240 (12.5%)	
Freeze-all strategy	216/248 (87.1%)	774/914 (84.7%)		203/240 (84.6%)	2,102/240 (87.5%)	
Transferred embryos (*n*)	1.6 ± 0.5	1.9 ± 0.4	**<0.001^a^**	1.6 ± 0.5	1.7 ± 0.5	*0.781^a^*
Good-quality embryos			0.054^b^			*0.920 ^b^*
Yes	143/248 (57.7%)	588/914 (64.3%)		171/240 (71.3%)	170/240 (70.8%)	
No	105/248 (42.3%)	326/914 (35.7%)		69/240 (28.7%)	70/240 (29.2%)	
Endometrial thickness before FET	10.5 ± 2.0	10.5 ± 2.0	0.610^a^	10.6 ± 2.0	10.6 ± 2.1	*0.722**^a^***

*Data are presented as mean ± SD, n (%) or median (IQR)*.

a *t-test for Equality of Means*.

b *χ^2^-test*.

c *Independent-Samples Mann-Whitney U-Test*.

*PSM, Propensity score matching; NC, natural cycle; mNC, modified natural cycle; BMI, body mass index; IVF, in vitro fertilization; ICSI, intracytoplasmic sperm injection; bFSH, basic follicle stimulating hormone; FET, frozen-thawed embryo transfer*.

*P < 0.05 indicated statistically significant differences between groups*.

### Pregnancy Outcome Measures

As demonstrated in Table 2, between-group comparisons in both mNC and NC group revealed significant differences in positive pregnancy rate (PPR), clinical pregnancy rate (CPR) and live birth rate (LBR) after PSM. More specifically, following the PSM analysis, CPR was 40.4% following NC-FET and 29.6% following mNC-FET (*P* = 0.013). LBR was 27.5% for mNC-FET vs. 36.3% for NC-FET (*P* = 0.040). PPR was 38.3% for mNC-FET vs. 48.3% for NC-FET (*P* = 0.027). No significant differences were detected between the two groups in terms of biochemical and clinical pregnancy loss rate and ectopic pregnancy rate (all *P* > 0.05).

**Table 2:** Pregnancy outcomes of FET cycles before and after PSM.

**Outcomes**	**Before PSM**			**After PSM**		
	**mNC (*N* = 248)**	**NC (*N* = 914)**	***P*-value^a^**	**mNC (*N* = 240)**	**NC (*N* = 240)**	***P*-value^a^**
Positive pregnancy rate (*n*, %)	96/248 (38.7%)	440/914 (48.1%)	**0.008**	92/240 (38.3%)	116/240 (48.3%)	**0.027**
Clinical pregnancy rate (*n*, %)	72/248 (29.0%)	370/914 (40.5%)	**0.001**	71/240 (29.6%)	97/240 (40.4%)	**0.013**
Live birth rate, LBR (*n*, %)	68/248 (27.4%)	344/914 (37.6%)	**0.003**	66/240 (27.5%)	87/240 (36.3%)	**0.040**
Biochemical pregnancy loss (*n*, %)	21/96 (21.9%)	57/440 (13.0%)	**0.025**	19/92 (20.7%)	16/116 (13.8%)	0.189
Clinical Pregnancy loss rate (*n*, %)	4/72 (5.6%)	26/370 (7.0%)	0.801	5/71 (7.0%)	10/97 (10.3%)	0.463
Ectopic pregnancy rate (*n*, %)	3/96 (3.1%)	13/440 (3.0%)	1.000	2/92 (2.2%)	3/116 (2.6%)	1.000

a *χ^2^-test*.

*PSM, Propensity score matching; NC, natural cycle; mNC, modified natural cycle; FET, frozen-thawed embryo transfer*.

*P < 0.05 indicated statistically significant differences between groups*.

A binary logistic regression model was also used to assess the association between endometrial preparation protocols and pregnancy outcomes while adjusting for potential confounders (Table 3). In the adjusted models, NC-FET was associated with an increased likelihood of clinical pregnancy and live birth compared with mNC-FET [OR 95% CI 0.66 (0.48–0.92), *P* = 0.014; OR 95% CI 0.71 (0.51–0.98), *P* = 0.039, respectively].

**Table 3:** Relationship between endometrial preparation and pregnancy outcomes in different models.

**Pregnancy outcomes**	**Endometrial preparation**	**Crude model^a^**		**Adjusted model^b^**	
		**OR (95% CI)**	***P*-value**	**OR (95% CI)**	***P-*value**
Positive pregnancy	NC	Reference		Reference	
	mNC	0.68 (0.51–0.91)	**0.008**	0.76 (0.56–1.03)	0.077
Clinical pregnancy	NC	Reference		Reference	
	mNC	0.60 (0.44–0.82)	**0.001**	0.66 (0.48–0.92)	**0.014**
Live birth	NC	Reference		Reference	
	mNC	0.63 (0.46–0.85)	**0.003**	0.71 (0.51–0.98)	**0.039**

*BMI, body mass index; FSH, follicle stimulating hormone; OR, odds ratio; CI, confidence interval; mNC, modified natural cycle; NC, natural cycle; FET, frozen-thawed embryo transfer*.

a *No adjustments for other covariates*.

b *Adjusted for female age (<37 years, ≥37 years), infertility duration, gravidity, parity, infertility type (primary, secondary), BMI (<25, ≥25 kg/m^2^), basic FSH (<10, ≥10 UI/L), initial treatment (IVF, ICSI), number of oocytes retrieved (≤ 3, 4–9, 10–15, >15), total number of embryos (<3, ≥3), number of transferred embryos, protocol in fresh cycle (fresh embryo transfer, freeze-all strategy), good-quality embryos (yes, no) and endometrial thickness before FET*.

*P < 0.05 indicated statistically significant differences between groups*.

## Discussion

To our knowledge, just a few studies have examined the various methods in which endometrium is prepared in women who have regular menstrual cycles. In contrast to previous studies, our practice can provide evidence-based recommendations about the optimal endometrial preparation procedures for FET, based on *post-hoc* randomization and a large sample size. We evaluated two distinct endometrial preparation protocols for frozen embryo transfer with NC and mNC in this retrospective cohort study. The results indicated that the mNC-FET protocol may have a decreased incidence of LBR than the NC-FET protocol in women with regular menstrual cycles.

In this study, all the patients of the NC and the mNC had regular menstruation and normal ovulation, and the intimal transformation and endocrine changes of patients with natural cycle were closer to the natural physiological state. If we can accurately grasp the opportunity of “implantation window,” the clinical outcome should be the best. Some studies have shown that hCG trigger has an adverse effect on clinical pregnancy rate in modified natural cycles (26, 27). Thus, it is an important question to consider whether it is appropriate to add hCG trigger in the modified natural cycle. In a pilot study, researchers found that LH elevation ≥13 mIU/ml prior to hCG administration may negatively affect clinical pregnancy rates in mNC for single euploid blastocyst transfer (27). Similarly, Montagut et al. indicated that natural cycles with a spontaneous LH peak had a better reproductive success when compared to modified natural cycles (28). This discrepancy may be explained by the fact that certain individuals with modified natural cycles have elevated LH on the day hCG is triggered, which is associated with decreased pregnancy rates.

A number of investigations on the clinical pregnancy outcomes of various endometrial preparations have been reported, however the findings have been inconsistent. In a retrospective study in 2016, patients with spontaneous LH peaks in natural cycles had better reproductive outcomes than modified natural cycles (28). Another study concluded that natural endometrial preparation yielded better outcomes in comparison to exogenous hormonal replacement cycles (9). However, there are also a lot of studies show no statistically significant differences in clinical outcomes between different protocols (29–31). Several studies have shown that endometrial thickness is a positive predictor of clinical outcomes (32, 33). Recently, one study showed endometrial thickness on the day of progesterone administration in natural cycles was thinner than modified natural cycles (34). This may have adverse effects on pregnancy outcomes of FET in natural cycles. Luteal support was not used in the study of Mackens et al. which could have had a negative impact on clinical pregnancy outcomes. These may be the reasons for the different results (30, 35).

In the endometrium of ovulation induced by hCG, the priority location of LH/CG receptor (LHCGR) has changed. These results indicate that exposure to hCG for ovulation induction impairs epithelial LHCGR staining in human ART endometrium, but the staining around the endometrial spiral arteries appears enhanced. Notably, maximum luminal epithelial LHCGR expression occurs during the early-mid secretory phase of the natural cycle, suggesting that the endometrium is best prepared to react to embryonic signals (36). In conclusion, the downregulation of LHCGR on the endometrial glandular epithelium following chronic exposure to hCG may help explain why the adoption of an hCG trigger to induce ovulation in a modified natural cycle is detrimental to implantation (37, 38). Furthermore, Andersen et al. discovered that the early luteal phase is hormonally abnormal and distinct from the conditions observed during the natural menstrual cycle: the timing of the hCG and progesterone rise is significantly faster following an hCG trigger than during the natural menstrual cycle, and the timing of the peak progesterone concentration is significantly advanced following an hCG trigger. Collectively, the endometrium is likely to advance after an hCG trigger, reducing the likelihood of optimum implantation (39).

In our current study, there are several remarkable aspects. Firstly, the statistical capacity is guaranteed because of the large sample size. Secondly, to make the results more reliable, we adjust more variables. Thirdly, statistical methods are reasonable. Last but not least, our study is focused on real clinical data and not on radical trials, thus avoiding strict inclusion criteria and exclusion criteria which may restrict studies' representativity and authenticity. There are some drawbacks to this study. To begin, we are unable to examine other confounding variables, such as exercise, nutritional supplements, or food, since this is a retrospective investigation. Second, since this is not an RCT, patients are allocated to different groups, which may introduce selection bias. mNC-FET may be as a way to improve, at least partially, control over the planning of FET operations.

However, we use PSM to control the confounding factors between the two groups. Thirdly, since cleavage stage embryo transfer is still a popular procedure in our clinic, the effectiveness of FET in natural cycles for patients undergoing blastocyst transfer requires further studies to be verified. Finally, it is necessary to consider the cancellation of multiple FET cycles due to no dominant follicle development or luteinization of unruptured follicles in the NC plan, prolonging the time of pregnancy and frequent visits, including ultrasound examination and blood sampling, which may be unfriendly to patients.

## Conclusion

Our findings show that the NC-FET without the hCG trigger had a higher incidence of clinical pregnancy and live birth than the mNC-FET. Therefore, caution is required while administering NC-FET to prevent hCG triggering. However, further prospective randomized clinical trials are expected to validate the NC-FET protocol's benefits. These research will result in improved counseling and management of patients with FET.

## Data Availability Statement

The datasets presented in this study can be found in online repositories. The names of the repository/repositories and accession number(s) can be found below: https://doi.org/10.6084/m9.figshare.13641296.

## Ethics Statement

The studies involving human participants were reviewed and approved by Reproductive Ethics Committees of the Affiliated Hospital of Shandong University of TCM. The patients/participants provided their written informed consent to participate in this study. Written informed consent was obtained from the individual(s) for the publication of any potentially identifiable images or data included in this article.

## Author Contributions

Z-GS and J-YS conceived and designed the study and revised the manuscript for important intellectual content. D-DG, X-XW, LL, and YZ contributed to data collection. J-YS and D-DG performed the statistical analysis. D-DG and LL wrote the manuscript. All the authors analyzed, interpreted the data, and approved the final version of the manuscript.

## Conflict of Interest

The authors declare that the research was conducted in the absence of any commercial or financial relationships that could be construed as a potential conflict of interest.

## Publisher's Note

All claims expressed in this article are solely those of the authors and do not necessarily represent those of their affiliated organizations, or those of the publisher, the editors and the reviewers. Any product that may be evaluated in this article, or claim that may be made by its manufacturer, is not guaranteed or endorsed by the publisher.
